# Critical Determinants in ER-Golgi Trafficking of Enzymes Involved in Glycosylation

**DOI:** 10.3390/plants11030428

**Published:** 2022-02-04

**Authors:** Ning Zhang, Olga A. Zabotina

**Affiliations:** Roy J. Carver Department of Biochemistry, Biophysics, and Molecular Biology, Iowa State University, Ames, IA 50011, USA; ningz@iastate.edu

**Keywords:** glycosyltransferases, ER-Golgi trafficking, mechanism of protein sorting, COPI and COPII complexes, sequences and motifs involved in trafficking

## Abstract

All living cells generate structurally complex and compositionally diverse spectra of glycans and glycoconjugates, critical for organismal evolution, development, functioning, defense, and survival. Glycosyltransferases (GTs) catalyze the glycosylation reaction between activated sugar and acceptor substrate to synthesize a wide variety of glycans. GTs are distributed among more than 130 gene families and are involved in metabolic processes, signal pathways, cell wall polysaccharide biosynthesis, cell development, and growth. Glycosylation mainly takes place in the endoplasmic reticulum (ER) and Golgi, where GTs and glycosidases involved in this process are distributed to different locations of these compartments and sequentially add or cleave various sugars to synthesize the final products of glycosylation. Therefore, delivery of these enzymes to the proper locations, the glycosylation sites, in the cell is essential and involves numerous secretory pathway components. This review presents the current state of knowledge about the mechanisms of protein trafficking between ER and Golgi. It describes what is known about the primary components of protein sorting machinery and trafficking, which are recognition sites on the proteins that are important for their interaction with the critical components of this machinery.

## 1. Introduction

All living cells generate structurally complex and compositionally diverse spectra of glycans and glycoconjugates, critical for organismal evolution, development, functioning, defense, and survival. The glycans attached to proteins and lipids determine their activity, solubility, subcellular localization, and structural organization in cells during normal and stressed conditions. Glycan-rich cell walls control cell growth and morphogenesis and protect them against environmental stresses. Glycosylation is the reaction that forms glycosidic linkages between activated sugar (donor substrate) and acceptor substrate (protein, lipid, polysaccharide, etc.). This reaction is performed by a large group of specialized enzymes, called glycosyltransferases, and broadly takes place in most organisms, such as yeast, humans, plants, etc. Because of the diversity of sugars and acceptor substrates, the resulting products of glycosylation present high variability and complexity in structures and functions.

Glycosyltransferases (GTs) are distributed among more than 130 gene families [1]. Most GTs are type II transmembrane proteins with several distinct domains: a short *N*-terminal cytosolic tail, transmembrane domain (TMD), flexible stem region, and large catalytic domain. Another group of GTs comprises integral membrane proteins with multiple TMDs and a large catalytic domain frequently localized on the cytosolic side of the membrane. Based on the type of catalytic domain folds, the GTs are grouped into GT-A, GT-B, and GT-C [2,3]. Two tight β/α/β Rossmann domains form a central *β*-sheet in GT-A folding, and mostly GT-A type proteins contain DxD catalytic motifs that cooperate with metal. Instead of being tightly associated, the two β/α/β Rossmann domains in GT-B are separate and form a cleft. GT-C proteins are predicted and found based on sequence and structure research and contain multiple hydrophobic helices. In addition, it was also found that the structure of the peptidoglycan glycosyltransferase of Aquifex aeolicus contains a lysozyme-like domain [4].

The primary types of glycosylation in various glycoconjugates are *N*-glycosylation and *O*-glycosylation. *N*-glycosylation is the formation of the glycosidic linkage between the amino group of an asparagine residue and the first sugar of the glycan. Asparagine in the Asn-X-Ser/Thr consensus sequence is the candidate for *N*-glycosylation, although not all Asn residues are glycosylated [5]. *N*-glycosylation is essential and usually impacts protein solubility, structure, and folding. It is also essential for protein localization and interactions with glycan-binding proteins. The glycan oligosaccharides in *N*-glycosylated proteins share the core sugar sequence and structure-Manα1-6(Manα1–3)Manβ1-4GlcNAcβ1-4GlcNAcβ1-Asn [6], which can be further branched with different sugars depending on the type of mature glycan synthesized. The *N*-glycosylation reactions take place in the endoplasmic reticulum (ER) and Golgi. The initiation of *N*-glycosylation occurs in the ER with the biosynthesis of the precursor oligosaccharide [7], which is later transferred to the peptide’s Asn by the oligosaccharyltransferase (OST) complex.

At the end of the multistep process, the glycoprotein is transported to cis-Golgi, and the next steps of *N*-glycan processing continue in different Golgi cisternae. The GTs, such as medial-Golgi-localized *N*-acetylglucosaminyltransferase I (GnTI) [8,9], GnTII [10,11], trans-Golgi-localized Galactose-1-phosphate uridylyltransferase l (GALT) [12], *β*-galactoside-α2,6-sialyltransferase l (ST6Gal-l) [13,14,15], and *β*-galactoside-α2,3-sialyltransferase-III (ST3Gal-llI) [16,17] are localized in distinct Golgi cisternae and responsible for subsequent steps of the synthesis of final glycan structures. It was demonstrated that most of these GTs form homo- and heterocomplexes, most likely to support the error-proof synthesis [14,18,19].

The *O*-glycosylation process is different in plant and mammalian cells. In mammalian type *O*-glycosylation, the glycan is attached to the hydroxyl group of the serine or threonine residue in a glycoprotein. *O*-glycosylation primarily occurs in Golgi and is also found in the cytoplasm and nucleus [20,21,22]. The first sugar, *N*-acetylgalactosamine (GalNAc), links to serine and threonine residues in Golgi and is called *O*-GalNAc. Multiple core sugar sequences are found in *O*-GalNAc-type glycosylation, and different biosynthesis steps are involved [6,23]. In plants, the main *O*-glycoproteins are the hydroxyproline-rich glycoprotein (HRGP) superfamily, including arabinogalactan proteins (AGPs), extensions (EXTs), and the repetitive Pro-rich proteins (PRPs) [24]. The type II arabino-3,6-galactans (AGs) get attached to noncontiguous Hyp residues in AGPs in Golgi [24]. In *A.thaliana*, eight Hyp-galactosyltransferases added the Gal to Hyp residues as the initiation steps of *O*-glycosylation of AGPs [24,25]. The variation of AGPs depends on the complexity of galactan side chains attached to Hyp residues [24]. The *β* 1-6 galactosyltransferases are involved in the elongation of the side-chain backbone; other GTs, such as arabinosyltransferases, rhamnosyltransferases, and xylosyltransferases, further branch this galactan backbone [24]. The Ser residues and three to five contiguous Hyp residues are the candidate sites of *O*-glycosylation in EXTs. The *β* 1-3-arabinosyltransferase attaches the first arabinofuranose reside to Hyp [24]. The Reduced Residual Arabinose 1–3 (RRA1-RRA3), Xyloglucanase 113 (XEG113), and Extensin Arabinose Deficient (ExAD) add the second, third and fourth Araf residues sequentially [24].

Cell-wall polysaccharides are synthesized in two locations: the Golgi and the plasma membrane. On the plasma membrane, most GTs involved in synthesizing polysaccharides are integral membrane proteins with multiple TMDs. For example, in plants, cellulose synthases (CESA) [3,26,27,28] are organized in multiprotein cellulose synthase complexes (CSCs) and synthesize cellulose microfibrils. These complexes are assembled in Golgi and delivered to the plasma membrane via cargo carriers. Multiple isoforms of CESA are identified: in primary plant cell wall synthesis, CESA1, CESA2, CESA3, CESA5, CESA6, and CESA9 assemble CSCs in Golgi [28,29]. At the same time, CESA4, CESA7, and CESA8 are involved in the biosynthesis of the secondary plant cell wall [28,29]. Other plant cell wall polysaccharides representing pectins and hemicelluloses are synthesized in Golgi by Golgi-localized GTs [3]. The latest studies suggest that Golgi-localized GTs are also organized in multiprotein complexes to synthesize polysaccharides. For example, *β* -1,4-xylosyltransferase (IRX) 9, IRX10, and IRX14 are xylosyltransferases and form the protein complex to synthesize the backbone of xylan [30,31,32]. The other seven GTs (cellulose synthase-like C4; xyloglucan xylosyltransferases XXT1, XXT2, and XXT5; galactosyltransferases XLT2 and MUR3 and fucosyltransferase FUT1) involved in xyloglucan biosynthesis were also shown to form heterocomplexes [3,33,34]. The homogalacturonan synthesizing galacturonosyltransferase (GAUT) 1 and GAUT7 proteins form a heterocomplex required to anchor catalytically active GAUT1 to Golgi [35].

## 2. Main Components of the Secretory Pathway

The trafficking of enzymes involved in glycosylation is essential for their proper delivery to the sites of their functioning in the cell, but it is still poorly understood. However, the information about trafficking processes available for various other proteins can also be applied to GTs. More information has recently become available concerning the primary components and routes of secretory pathways. The GTs and glycosidases involved in glycosylation, the focus of this review, most likely follow a similar secretory pathway and therefore are subjected to similar sorting mechanisms like other membrane proteins.

**Figure 1:**
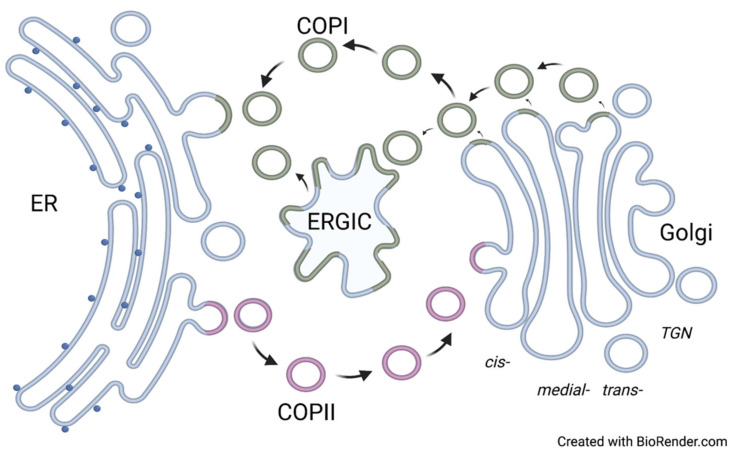
The cargo transportation between ER and Golgi via COP-coated cargo carriers. ER: endoplasmic reticulum; COPI: coat protein complex I; COPII: coat protein complex II; ERGIC: ER-Golgi intermediate compartment. Note that the ERGIC compartment has not been demonstrated in plant and yeast cells.

Thus, it has become clear that the coat protein complex I (COPI) -coated cargo carriers and the coat protein complex II (COPII)-coated cargo carriers mediate the trafficking path between the ER and the Golgi (Figure 1). One part of this trafficking pathway is the sorting signal in the protein sequence of GTs, which is recognized by the cargo receptor or COP coatomer to trigger the trafficking of proteins. The transmembrane region and *N*-terminal cytoplasmic domain of lipid phosphatase Sac1 are essential in the retention mechanism of phosphatidylinositol-3-phosphatase (Sac1) in Golgi [36]. The core components of the COPI-coated cargo carriers are the *α*-COP, *β*-COP, *β’*-COP, *γ*-COP, *δ*-COP, *ε*-COP, and *ζ*-COP subunit. The activation of the GTPase ADP-ribosylation factor 1 (Arf1) is a prerequisite for the assembly of the COPI coat [37,38,39]. Guanine nucleotide-exchange factors (GEFs) stimulate the GTPase Arf1 activation by exchanging GDP to GTP; then, the activated GTPase Arf1 embeds into the lipid membrane by using a myristoylated α-helix [40,41]. In turn, GTPase Arf1 recruits the coatomer complex with an inner coat and outer coat to transport protein and lipid cargo from the Golgi to the ER and between the Golgi cisternae [42,43]. COPII-coated cargo carriers deliver the cargo proteins from the ER to the Golgi, with the inner and outer layers of the protein lattice acting as the core component of COPII-coated cargo carriers [44,45,46,47]. The activation of the Ras-like small COPII coat Sar1 GTPase is the prerequisite of the assembly of the COPII coat [48]. Guanine nucleotide-exchange factor Sec12 assists the exchange of the GDP to the GTP on Sar1 to recruit the coat protein Sec23-Sec24 inner layer of the protein lattice, and the heterotetramers of coat protein Sec13 and Sec31 as the outer layer of the protein lattice is recruited to continue the assembly of the COPII coat [44,45,46,47].

There are some differences in COPI and COPII transportation in plants compared to animals and yeast. In plant cells, the COPII and COPI-coated cargo carriers’ transportation impacts plant growth, stress response, and protein transportation. In *A.thaliana*, multiple paralogs of COPI components have been discovered [49,50]. The silencing of the *β*1/2-COP gene enhanced the sensitivity of *A.thaliana* to salt stress [49,51]. Double mutant of *β*1/2-COP and a single mutant of *α*2-COP were dwarfed compared to *Col-0* [49,52], and the silencing of *β*1/2-COP or *α*2-COP or p24 protein altered the structure of the Golgi [49,52,53]. The knockout of *α*1-COP, *γ*-COP, and *ε*-COP resulted in a reduction in seed production due to altered pollen grain adherence and pollen tube germination [54]. The *β*′-, *γ*-, and *δ*-COP proteins were shown to interact with each other and were localized in Golgi [55]. The *β*′-, *γ*-, and *δ*-COP proteins were required to support the Golgi structure, and the silence of these genes caused the plant cell death [55]. The maintenance of the Golgi structure also requires the recruitment of Arf1 [56] and multiple paralogs of the cargo receptor p24 family protein [53]. The multiple paralogs of the p24 family proteins are involved in the trafficking of GPI-anchored proteins to the plasma membrane. For example, it was shown that these paralogs could interact with GPI-anchored protein arabinogalactan protein 4 [50]. In the *Arabidopsis p24δ3δ4δ5δ6* quadruple mutant, ER lumen protein-retaining receptor A(ERD2a), as the K/HDEL receptor, accumulated in Golgi and, as a result, the expression of *Sec31* gene was upregulated [53]. The p24 family protein affected the ERD2a trafficking by direct interaction with ERD2a via luminal GOLD domain, and their interaction showed pH-dependence [57,58].

The Multiple paralogs of COPII protein components were also discovered in plants [59]. The expression pattern, subcellular localization, and function of the COPII protein paralogs showed a significant difference [60,61,62,63]. SEC23A/SEC23D [64], SEC31A/B [61,65] and Sar1B/C [62] had been reported to be required in pollen development. In *Physcomitrium patens*, Sec23D is localized in the presumptive ER-exit sites [60]. The knockout of the *Sec23d* gene suppressed the protein transportation between ER and Golgi and protein secretion in the mutant plants, leading to ER morphology defects and ER stress [60]. In *A.thaliana*, Sar1A is also localized in ER-exit sites [63]. The residue Cys84 in the Sar1A is crucial for its specific interaction with AtSec23A, essential for the ER-export process [63]. The mutation of this residue disturbed their interaction resulting in the suppression of the ER-export process of vacuolar protein [63]. In *A.thaliana*, the formation of unusually giant COPII vesicles, which modulated the transport of channel proteins and transporters, was observed in response to stress conditions [66]. Even though the importance of COPII components in plant growth and development has been demonstrated, the existence of COPII-coated cargo carriers in plant cells is still disputed [67,68]. The notion that the Golgi entry core compartments can work independently from COPII-coated cargo carriers formation has been proposed [69].

The cargo sorting during COPI and COPII coat assembly is essential. The sorting signal in the cargo protein plays a dominant role in the direct or indirect interaction between cargo proteins and components in the coat. The Sec24 subunit in the COPII complex is involved in cargo sorting and directly binds with the cargo protein [70,71,72]. The diverse cargo binding sites in the Sec24 protein recognize different cargo sorting signal motifs in cargo proteins, and multiple isoforms of Sec24 are involved in diverse cargo sorting [71,72,73]. For example, the ER-exit signal DxE [74,75], LxxLE [75,76], YxxNPF [77], triple arginine (RRR) motif [78], and ΦXΦXΦ motif of bovine anion exchanger 1 (AE1) [79] are recognized by Sec24. Sar1 also participates in cargo sorting and directly interacts with cargo sorting signal motifs. Sar1A directly interacts with the polybasic motif of planar cell polarity protein Frizzled-6 to adjust the cargo packaging into coated cargo carriers [80]. The RNKR motif of Drosophila type I transmembrane protein Crumbs is the ER-exit signal interacting with Sar1 [81]. Cargo transportation via COPII-coated cargo carriers also requires the protein–protein interaction between cargo sorting motifs and COPI coatomers or cargo receptors. The KXD/E motif of *A.thaliana* endomembrane protein 12 binds with COPI coatomers [82]. *α*- COP, *β*’-COP, and *γ*-COP bind with diverse motifs to assemble the COPI-coated cargo carriers. For example, *α*- COP and *β*’-COP directly bind di-lysine motifs [83], and *γ*-COP recognizes the FFxxBB(x)n of the P24 protein [38] and directly binds with human ER *α*-1, 2-mannosidase [84].

Besides the direct interaction between the cargo protein and COP complex, the cargo receptors mediate the cargo sorting. In mammalian cells, the ER-Golgi intermediate compartment (ERGIC) ERGIC-53 protein [85] and p24 protein families [76,77,86] are known as the cargo receptors for the soluble cargo proteins, and Erv29 proteins are involved in COPII vesicles assembly with the GFP-HDEL and glycosylated pro–α-factor (gpαf) cargo proteins [87,88,89]. Transmembrane proteins Erv14 [90,91] and Erv26 [72] work as the cargo receptors for the membrane proteins via their direct interaction with Sec24 and cargo proteins. It has been shown that KDEL receptors recognize ER-retrieval signal KDEL and directly interact with the KDEL motif. At the same time, the strength of interaction and release of cargo protein is regulated by the difference of pH in the ER and the Golgi [72]. The Arg residues in the KDEL receptor anchored the KDEL peptides via salt bridge interactions, whereas the Glu residues formed a hydrogen bond with tryptophan in the KDEL receptor [92]. The Glu residues hydrogen bonding with histidine in the KDEL receptor was shown to be pH-sensitive [92]. The cargo receptor Rer1 binds to the KKXX motif in the ER membrane proteins [93] and the polar residue in the TMDs [94]. The positively charged amino acids in the cytosolic tail, such as the di-arginine motif (RXR) and di-lysine motif (KK or KXKXX), have proven to play essential roles in the trafficking of type I membrane proteins [38]. In addition, so-called kin recognition has also been proposed. The interactions between kin oligomers and GTs and glycosidases occur in specific Golgi cisternae and prevent their entry into transportation carriers and their forward movement to later cisternae [95].

## 3. Specific Sequence Motifs Involved in GTs and Glycosidases Sorting and Trafficking

**Figure 2:**
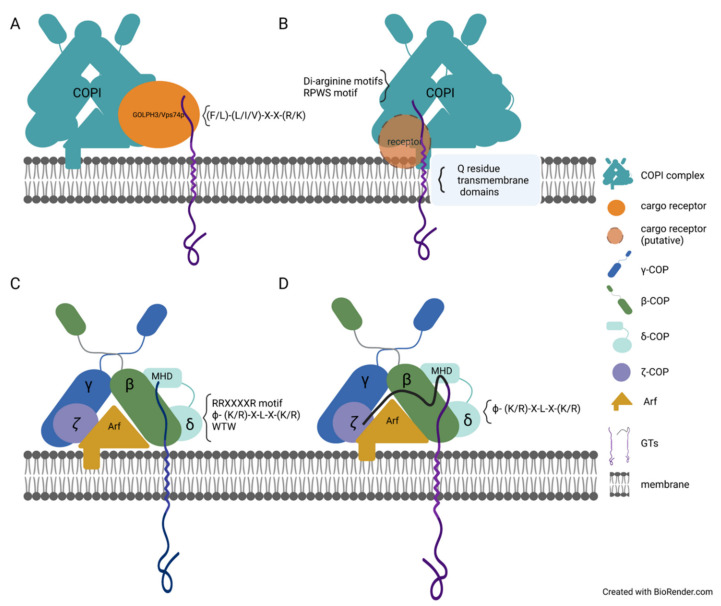
The protein–protein interaction between cargo sorting signal motifs in the GTs and COPI coatomers or cargo receptors. (**A**): The cargo sorting signal motifs in the cytosolic tail of GTs interact with cargo receptors (e.g., Vps74p and GOLPH3). (**B**): The GTs interact with putative cargo receptors or directly interact with the COPI complex via cargo sorting signal motifs in the cytosolic tail and/or TMDs. (**C**): The cargo sorting signal motifs in the cytosolic tail of GTs interact with the MHD domain of *δ*-COP and *β*-COP. (**D**): The cargo sorting signal motifs in the cytosolic tail of GTs interact with the MHD domain of *δ*-COP, *ζ*-COP, and *β*-COP. The figures are created in BioRender.com.

The mechanism of trafficking GTs and glycosidases between the ER and the Golgi is somewhat similar to the mechanism of diverse protein sorting in the Golgi and ER. The transportation and localization of GTs and glycosidases rely on the presence of a single amino acid (e.g., arginine, lysine, leucine, and phenylalanine residues) in the cytosolic tails of GTs, or several amino acids together determine the localization of GTs. In yeast, the consensus sequence of (F/L)-(L/I/V)-X-X-(R/K) is broadly found in many GTs and has been shown to interact with the cargo receptor Vps74p protein in the assembly of COPI-coated cargo carriers (Figure 2A) [96]. The single mutation of F4 or L5 and the double mutation of K7 and R8 in the FLSKR motif in the cytosolic tail of α 1,2-mannosyltransferase (Kre2p) impaired the protein interaction between vacuolar protein sorting-associated protein 74 (Vsp74) and Kre2p. Thus, the presence of the F4, L5, K7, and R8 residues is required in the Kre2p cargo transportation via the coat complex COPI-coated cargo carriers [96]. In plants, the localization of *A.thaliana* ER-*α*-mannosidase I (MNS3) depends on the four amino acid sorting signal. MNS3 typically localizes in the early Golgi, and the LPYS Golgi-targeting signal motif in the cytosolic tail of MNS3 is believed to be involved in the retaining mechanism [97]. The fusion protein MNS3-GFP-HDEL contained the GFP and ER-targeting signal HDEL at the C terminal of MNS3 and was primarily localized in the Golgi and weakly in the ER [97]. This indicated that the retrieval function of the ER-target signal HDEL is inhibited in this fusion protein, and the LPYS Golgi-target signal drives the localization of MNS3. However, the deletion of the LPYS Golgi-target signal and, specifically, the mutation of leucine in the LPYS motif recruited fusion protein, MNS3-GFP-HDEL, to the ER [97], which demonstrates the LPYS Golgi-target signal and primarily the leucine residue is essential for localization of MNS3.

The arginine and lysine residues also play a dominant role in the transportation of GTs in plants and animals. Di-arginine motifs (RR, RXR, or RXXR) are other ER-retaining signals in plants [98]. The first 90 amino acids have been proved to be sufficient to localize *A.thaliana* glucosidase I (AtGCSI) to the ER [99]. The di-arginine motifs (R6R7SAR10GR12) were found in the cytosolic tail of AtGCSI, and the mutation of all four arginine residues altered its ER-localization to the Golgi. In contrast, preserving only one of these di-arginine motifs was sufficient for retaining the AtGCSI protein in the ER and punctate structures [98]. In animals, three isoforms of GM3 synthase (SAT-I), a GT involved in ganglio-series ganglioside synthesis, were discovered and named M1-SAT-I, M2-SAT-I, and M3-SAT-I. Surprisingly, these three isoforms showed different localizations: M2-SAT-I and M3-SAT-I were localized in the Golgi, and M1-SAT-I was found in the ER [100]. The arginine-rich motif RRXXXXR has proven critical for M1-SAT-I retention in the ER. The single mutation of any arginine in this motif could not change the ER-localization of M1-SAT-I. However, the mutation of any two arginine residues in this arginine-rich motif held M1-SAT-I in the Golgi [100].

In addition to being essential for the ER retention of GTs, the arginine and lysine residues are also involved in the Golgi-targeting of GT and glycosidases in both plant and animal cells. In *N. tabacum*, two arginine residues were found in the cytosolic tail (MR2GYK5FCCDFR11) of Golgi-localized *N. tabacum* GnTI [101]. The mutation of R11 and K5 resulted in GnTI being localized in the ER and the Golgi, while the mutation of R11, K5, and R2 held GnTI predominantly in the ER. However, the mutation of K5 and R2 resulted in GnTI being localized only in the Golgi [101]. These results demonstrate that the arginine residues (R11) proximal to the TMD are essential for retaining GnTI in the Golgi [101]. The lysine residues in the cytosolic tail (MPRKRTLVVN) of *A. thaliana* a-mannosidase II (GMII) are required for Golgi-localization. In contrast, the arginine and lysine residues in the cytosolic tail (MSKRNPKILK) of *A. thaliana* glycosyltransferase XylT are essential for this protein localization in the Golgi [101].

In mammalian cells, the [RK](X)[RK] sequence, as an ER-exit signal, is found in the cytosolic tail of Golgi-localized *β*-1,3-galactosyltransferase (GalT2) [102]. GalT2 was localized in the ER when the RR motif was mutated [102]. Meanwhile, the replacement of RR to RAR/KAK/KK also held GalT2 in the Golgi, which indicates that the motifs with similar [RK](X)[RK] properties have similar functions [102]. The mutations in the [RK](X)[RK] sequence in the cytosolic tails of *β*-1,4-*N*-acetyl-galactosaminyltransferase (GalNAcT), GM3 sialyltransferase (Sial-T2), and *β*1,4Galactosyltransferase (*β*1,4GT) also altered their Golgi-localization [102]. Meanwhile, there are two [RK](X)[RK] motifs in the cytosolic tail of Golgi-localized Sial-T2, and the mutation of any [RK](X)[RK] motif resulted in the ER-localization of Sial-T2 [102]. The mutation of R7R8 held Sial-T2 in dual localization—the majority of the Sial-T2 proteins localized in Golgi and partial Sial-T2 protein localized in the ER. In contrast, the mutation of R23R25 held Sial-T2 mostly in ER and partially in the Golgi [102]. This indicated that the contribution of the [RK](X)[RK] motif to localization could differ. The conserved sequence “*ϕ*-K4LLQR8” was critical for the Golgi-localization of GlcNAc-1-phosphotransferase (Ptase). The localization of mutant proteins with the single mutations of the K4/R8/S15 residues did not overlap with the Golgi marker, GOLPH4 [103]. The Golgi-retention signal motif RPWS, which is in the cytosolic tail of UbiA prenyltransferase (UBIAD1), determines the protein localization in the Golgi, and the RPWS signal is highly conserved in its orthologs in different species [104]. The UBIAD1 protein with a mutation of the arginine residue in its RPWS motif failed to retain UBIAD1 in the Golgi [104]. The positive charge and branched structure of the arginine and lysine residues may be significant in the trafficking of GTs. Hence, the sorting signal in the cytosolic tail of GTs determines the localization of GTs.

Arginine-based motifs are broadly involved in the trafficking of GTs and retaining them either in the Golgi or ER. Therefore, the mechanism of recognizing arginine-based motifs as specific to ER or Golgi-target signals is unclear. The distance between the arginine-based motif and lipid bilayer and the distance between the arginine/lysine residues within the motif can affect the function of arginine-based motifs. For example, the ER-localized M1-SAT-I proteins changed localization primarily to Golgi when the amino acids 28-55 on its *N*-terminus were deleted. Such deletion shortened the distance between the ER-target signal RRXXXXR and membrane from 53 amino acids to 25 amino acids, indicating that the function of ER-target signal RRXXXXR in localization of M1-SAT-I requires a long enough functional distance [100]. The change in the distance between the arginine residues within motifs can also alter the localization of plant and mammalian GTs. For instance, increasing the distance between two arginine residues in the cytosolic tail of ER-localized *A.thaliana* AtGCSI recruited this protein to the Golgi or partial Golgi [98]. In addition, shortening or elongating the distance between K4 and R8 in the cytosolic tail of Ptase switched normal Golgi-localization of the Ptase to ER-localization [103].

## 4. Other Protein Domains Essential for the Trafficking of Enzymes Involved in Glycosylation

Although the cytosolic tail of GTs and glycosidases is critical for their localization [98,101,102], the transmembrane and luminal domains also impact the plant GTs localization. For example, the lumen domain of *A.thaliana* AtGCSI, as well as its di-arginine motifs in the cytosolic tail, affects the ER-localization of AtGCSI; the shortening of the AtGCSI lumen domain results in a switch from its ER-localization to Golgi-localization when the di-arginine motifs are deleted [98], which indicates the di-arginine motifs in the cytosolic tail and lumen domain independently affect the localization of AtGCSI. The *N*-terminus protein sequence of two proteins, Golgi-localized GnTI from *N. benthamiana* and trans-Golgi marker a–2,6-sialyltransferase (ST), are grouped into three parts: the cytosolic tail, the TMD, and the stem region. To study the function of the cytosolic tail, the TMD, and stem region in localization of GnTI and ST, three parts of sequences of two proteins were switched in different types of recombination [105]. The GnTI-ST-GnTI fusion protein generated by swapping the TMD of GnTI with the TMD of ST was mislocalized and unable to function correctly. Conversely, the switching of the cytosolic tail and stem region of GnTI to the cytosolic tail and stem region of ST did not impact the localization of the fusion proteins in the Golgi [105], indicating that the TMD is more critical for the correct localization of GnTI (Figure 2B). The highly conserved sequence (FIYIQ) in the TMD of NtGnTI is responsible for the protein localization in the cis/medial-Golgi [106]. The Q residue in the FIYIQ sequence is conserved, and the mutation of Q25 altered the cis/medial-Golgi localization of NtGnTI to trans-Golgi. In addition, the NtGnTI-Q25A-GFP was detected in the vacuole and occasionally in the apoplast, indicating the secretion of full-length NtGnTI-Q25A or a degradation product [106]. It was also observed that the mutation of Q25 impacted the formation of the homodimer of NtGnTI [106]. In *A.thaliana* GnTI, the Q residue has a similar function. The AtGnTI protein with a mutation on Q23 failed to restore the process of complex *N*-glycans synthesis in the *gntI* mutant plants and was mislocalized to apoplast instead of Golgi [106]. Furthermore, AtGnTI-Q23A-GFP protein was hardly detected on the immunoblot, while its transcript level was comparable with the level of wild-type mRNA. It was also estimated that the half-life of the AtGnTI-Q23A mutant protein was much shorter in comparison with wild-type protein, indicating that AtGnTI-Q23A-GFP is not stable and degrades more quickly [106]. To investigate how the Q residue affects the localization of AtGnTI, the Q23 residue was replaced by either His, Leu, Glu, Tyr, Val, or Ser residues. Only AtGnTI-Q23H was localized in Golgi and showed a result similar to that of the wild-type AtGnTI function when expressed in mutant *A.thaliana gntI* plants [106]. It was determined that the stem region of GnTI contributed predominately to homomeric and heteromeric protein complex formation [105].

In some cases, either the cytosolic tail, TMD, or lumen domain alone cannot determine the localization of GTs, and the cooperation of two domains is frequently required. The cooperation of several GTs domains to determine their localization is broadly reported in mammalian cells, but not much is known about such cooperation in plants. Therefore, below, we describe what is currently known in animals. The polypeptide *N*-acetylgalactosaminyltransferases (GalNAc-T) are type II transmembrane proteins localized in the Golgi. Although GalNAc-T1, GalNAc-T2, GalNAc-T7, and GalNAc-T10 belong to the same GT family, the mechanism that supports their Golgi-localization is different [107]. For example, neither cytosolic tail, TMD, or luminal stem domain could determine the Golgi-localization of Gal-NAc-Ts. It has been shown that GalNAc-T1 and GalNAc-T2 proteins share a similar mechanism where the cooperation of their cytosolic tail and TMD are required to determine their localization. The cytosolic tail or luminal stem together with TMD recruit GalNAc-T7 to the Golgi, whereas the luminal stem and TMD are necessary for the Golgi-localization of GalNAc-T10 [107].

The *N*-acetylglucosamine-1-phosphotransferase (PT) complex is involved in the biosynthesis of mannose 6-phosphate; *α*-, *β*-, and *γ* -subunits are the essential components of the Golgi-localized PT complex [108]. The maturation of *α/β*-subunits requires cleavage of the precursor protein. Both versions of the *α/β* -subunits, precursor and cleaved, were detected in the Golgi. The cleavage of the precursor protein in the Golgi is necessary for the assembly of the PT complex, and the ER-export of the precursor protein of *α/β* -subunit is a prerequisite of cleavage of *α/β* -subunit in the Golgi [108]. Thus, the trafficking of precursor protein to the Golgi considerably affects the proper function of the PT complex in the biosynthesis of mannose 6-phosphate. There are four potential ER-export signals in the *α/β* -subunit precursor protein: di-leucine motif (L5L6) in the *N*-terminus and [RK]X[RK] motifs (K1236RK1238, R1242RR1244, and R1253IR1255) in the *C*-terminus. The double mutation of L5L6 to AA and R1253IR1255 to AAA caused the precursor protein to be recruited to the ER [108], indicating that the ER-export of *α/β* -subunit precursor protein requires two ER-export signals on its *N*- and C-termini. UBIAD1 is involved in the biosynthesis of vitamin K and CoQ10, and UBIAD1 has eight putative TMDs. The UBIAD1 *N*-terminal domain contains the Golgi-retention signal RPWS, which, together with the first two TMDs, is required for its Golgi-localization [104].

## 5. Recycling of Glycosyltransferase and Glycosidases Involved in Glycosylation

The transport cargo carriers (i.e., the COPI and COPII complexes) are critical for GTs recycling. It was shown in plants when the *N*-terminal domain of GnTI and Sar1p were co-expressed in *N. benthamiana* leaf epidermal cells, both proteins were co-localized in the punctate structure at ER-exit sites (ERES) [101]. However, a mutant version of GnTI, where basic amino acids within its cytoplasmic tail were mutated, was not able to recruit Sar1 to ERES, indicating that COPII proteins are involved in GnTI transport. Even though the studies about the transport of the plant GTs via the COPI and COPII complexes are limited, the results indicate that the mechanism of membrane proteins transport in plant and mammalian cells are similar. For example, the LxxLE motif functions as the ER-export signal in animals and plants [75,76,109]. Hence, the advanced knowledge about trafficking of GTs via COPI and COPII-coated cargo carriers in animal cells might offer some clues to the GTs transport via COPI and COPII complexes in plants. For example, the silencing of the coatomer subunits *δ*COP or *ε*COP results in the mislocalization of the Golgi-resident *A.thaliana* MNS3-GFP protein [97]. During the formation of the COPI and COPII-coated cargo carriers harboring GTs as cargo, indirect or direct interactions between GTs and COPI/COPII complex proteins were observed. For example, the Vps74p protein was detected as the intermediate protein in interaction with the COPI complex in yeast, and the knockout of Vps74p impacted the localization of Kre2p, Mnn2, Mnn9, and Ktr6 [96]. Vps74p was shown to bind to Sec26p (*β*-COP) and Ret2p (*δ*-COP) in in vitro experiments (Figure 2A) [96].

**Figure 3:**
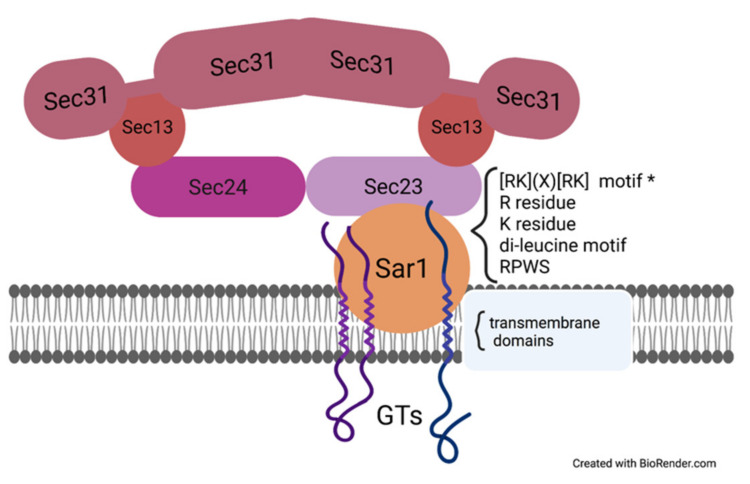
The protein–protein interaction between cargo sorting signal motifs in GTs and COPII coatomers.

In animal cells, the Vps74p ortholog protein GOLPH3 functions similarly to Vps74p in yeast. GOLPH3 binds to C2GnT and SiaTI in vitro, and all three proteins, GOLPH3, C2GnT, and SiaTI, were detected in COPI vesicles [110]. Meanwhile, the knockout of GOLPH3 triggered the mislocalization of C2GnT and SiaTI from ER/Golgi to Golgi only. At the same time, the content of C2GnT and SiaTI in COPI vesicles was significantly decreased [110]. In recent studies, GOLPH3 was proved to interact with not only the LxxR motif but the positively charged amino acids upstream of the LxxR motif, [111], which further confirmed the function of GOLPH3/ Vps74p in retaining the cargo protein in the Golgi cisternae and preventing cargo from leaving to the TGN [111,112]. The protein was transported to lysosomes when it escaped the GOLPH3-mediated cisternal inter-conversion mechanism. This indicated that GOLPH3/ Vps74p controls the lysosomal degradation of the protein [111,112]. The ER-target signal, R11R12XXXXR, in the cytosolic tail of M1-SAT-I has been proven to interact with *β*-COP or *δ*-COP (Figure 2C), while the mutation, M1-SAT-I-R11/12S, interrupted this interaction [100]. This indicates that the RR residues may directly bind to *β*-COP or *δ*-COP (Figure 2C). The Golgi protein GlcNAc-1-phosphotransferase (Ptase) synthesizes the mannose 6-phosphate recognition marker. The utilization of the recently developed BioID2 assay revealed the interactions among the Ptase, *δ*-, and *ζ*-COP subunit proteins [103]. The direct interaction between *δ*-/*ζ*-COP and Ptase was confirmed by pull-down assay, which also detected traces of *β*-COP and *γ*-COP (Figure 2D) [103]. It has been shown that Ptase directly binds to the highly reserved sequence, VRFSTE, in the MHD domain of *δ*-COP [103]. The mutations of K4 to Q, R8 to G, and S15 to Y in the cytosolic tail of Ptase impaired and weakened its interaction with *δ*-/*ζ*-COP [103]. The *ϕ*- (K/R)-X-L-X-(K/R) sequence is also found in the cytosolic tail of other GTs, such as C2GNT1, GALNT3, GALNT6, and GALNT8 [103]. C2GNT1, GALNT3, and GALNT8 directly bind to *β*-COP, *ζ*-COP, and the MHD domain of *δ*-COP (Figure 2D); GALNT6 interacts with *β*-COP and the MHD domain of *δ*-COP (Figure 2C) [103]. The arginine residues in the R3TLLR7R8R9 sequence in the cytosolic tail of C2GNT1 are essential for recruiting C2GNT1 to the Golgi. The mutation of arginine residues impaired the interaction between C2GNT1 and *ζ*-COP protein, and the interaction between C2GNT1 and the MHD domain of *δ*-COP (Figure 2D) [103]. In previous studies on CHO-K1 cells, C2GNT1 was shown to interact with GOLPH3 and later with COPI subunits [110]. However, in HeLa cells, the knockout of GOLPH3 did not affect the localization of C2GNT1 [103]. UBIAD1 is localized in the Golgi in L02 cells, but UBIAD1 is localized in the ER and the Golgi in both HEK293 and T24 cells [104], which indicates that the mechanism of trafficking of GTs might vary for different cell types. Although there is no *ϕ*- (K/R)-X-L-X-(K/R) sequence in the cytosolic tail of GALNT4, the WTW motif was found to be responsible for its interaction with the MHD domain of *δ*-COP and *β*-COP (Figure 2C) [103]. In addition, the Sar1 protein has been proven to interact with GTs directly. Synthetic cytosolic tails with RR motifs of GalNAcT and GalT2 interacted with Sar1 in vitro. The mutation of RR to AA impaired the interaction between Sar1 and GalT2 or GalNAcT (Figure 3) [102]. The cytosolic tails of GalNAcT and GalT2 bond to Sec23p in vitro, and the presence of active Sar1 increased interaction between GalNAcT or GalT2 with Sec23p [102].

In addition to Vps74p and GOLPH3 being shown to affect GT-localization via direct interaction with the COPI complex, other proteins are involved in determining the localization of GTs. The Golgi-localized STELLO1 and STELLO2 proteins (STL1 and STL2) from *A.thaliana*, which contain the glycosyltransferase-like domain, were shown to alter the CesA distribution and assembly via direct interaction with the latter [113]. The genes encoding the STL1 and STL2 proteins were co-expressed together with CesA genes in the *A.thaliana stl1stl2* mutant recovering the cellulose content that was reduced in the *stl1stl2* mutant [113]. In animal cells, the GlcNAcT-I inhibitory protein (GnT1IP) shares a similar protein sequence with GlcNAcT-IV glycosyltransferases and inhibits GlcNAcT-I activity [114]. Two GnT1IP transcripts were named GnT1IP-L and GnT1IP-S, and the GnT1IP-L protein was shown to be the type II membrane protein [114]. GnT1IP-L can interact directly with GlcNAcT-I, causing its mislocalization from the medial-Golgi to the ER, ERGIC, and cis-Golgi [114]. Golgi-resident GRASP55 regulated the subcellular localization of glycosylation protein involved in glycosphingolipid biosynthesis by direct interaction [115]. The L95LGV98 sequence in the GRASP domain of GRASP55 interacted with the cytosolic tail of GlcCer synthase (GCS), which catalyzes the critical step in glycosphingolipid biosynthesis [115]. The direct binding with GRASP55 promoted the correct subcellular localization of GCS by preventing GCS from entering in the retrograde transportation [115]. The GTP exchange factor GBF1 facilitated the phosphorylation of Arf1-GDP, and the Src tyrosine kinase (Src) played an essential role in the ARF GTP formation [116]. Src phosphorylated the Y876 and Y898 in the GEF domain C-terminus of GBF1, further increasing the binding between GBF1 and Arf1 and the GALNT relocation [116].

## 6. Protein–Protein Interactions Contribute to GT Trafficking

It was proposed that the protein–protein interactions between plant GTs are required for the ER-export of protein complexes. GAUT1 and GAUT7 are involved in plant cell wall pectin biosynthesis and form a protein complex in the Golgi [35]. The TMD of GAUT1 becomes post-translationally cleaved and, thus, the GAUT1-recruitments to the Golgi require its interaction with GAUT7 [35]. Proteomic analyses of wheat glycosyltransferases involved in the xylan synthesis [117] showed the network of protein–protein interactions among the glycosyltransferases TaGT43-4 and TaGT47-1, mutases TaGT75-3 and TaGT75-4, and the TaVER2 and TaGLP proteins. The protein–protein interactions among TaGT43-4, TaGT47-13, TaGT75-3, and TaGT75-4 were confirmed, and a single complex was detected via immunoblot analysis. TaGT43-4, TaVER2, and TaGLP were localized in the ER when transiently expressed in tobacco leaves, while TaGT47-13 was localized in trans-Golgi, overlapping with ST-GFP trans-Golgi maker [117]. Meanwhile, TaGT43-4 interacted with TaGT47-1, TaGLP, TaVER2, and TaGT75-4 to form heterodimers in the ER, and co-expression with TaGT47-13 recruited these heterodimers to trans-Golgi [117]. Thus, it is proposed that TaGT43-4 functions as a scaffold protein, assisting in forming a xylan biosynthesis complex in the ER [117]. It was proposed that the interaction between TaGT43-4 and TaGT47-13 was required for the xylan-synthesizing protein exportation from the ER to trans-Golgi [117].

In *Asparagus officinalis*, AoIRX14A and AoIRX10 proteins with catalytic DxD motifs are involved in xylan biosynthesis. AoIRX9, AoIRX14A, and AoIRX10 were detected within a single multiprotein complex via the GFP-trap approach [30]. When AoIRX9, AoIRX10, or AoIRX14A were individually expressed in *N.benthamiana* leaves, AoIRX9, and AoIRX10 localized in ER, while AoIRX14A localized in the ER and Golgi [30]. When AoIRX9, AoIRX14A, and AoIRX10 were co-expressed, the signal of AoIRX9-VENUS or AoIRX10-VENUS was detected in the Golgi, and direct protein interactions between AoIRX9 and AoIRX14A were confirmed [30]. These studies strongly suggest that AoIRX9, AoIRX10, and AoIRX14A function as components of the xylan-synthesizing complex, and the protein–protein interaction among these GTs is required for their ER-export.

It is also proposed that ER and Golgi acidic environments impact protein–protein interactions of GTs and, specifically, the formation of homo- and heterocomplexes. For example, the GnT-I, GnT-II, GalT-I, ST3Gal-III, and ST6Gal-I proteins form the homodimers in the ER, and no heterodimers among these GTs have been detected in the ER [118]. Meanwhile, the heterodimers among GTs were detected in Golgi, and the formation of heteromeric GT complexes inhibited the formation of homomers in the Golgi [118]. In the study by Antti Hassinen [119], treatment with chloroquine (CQ) altered the pH in the observed cells, thereby inhibiting the formation of heteromers. The increase in pH by 0.4 in the Golgi inhibited the formation of heteromers and facilitated the formation of homomers. Thus, the difference in pH between the ER and Golgi alters the probability of forming either heteromers or homomers [118].

## 7. Conclusions

The common mechanism of GTs and glycosidases trafficking between the ER and Golgi involves the motifs or specific amino acids in their cytosolic tails, their TMDs, and catalytic domains. These different factors can act either independently or cooperatively via directly or indirectly interacting with COP coatomer proteins, ultimately affecting the localization and transportation of GTs and glycosidases (Table 1). The common mechanism of GTs and glycosidases trafficking is similar to the trafficking mechanism of other proteins. Thus, the studies of other protein trafficking mechanisms can offer clues to investigate the potential mechanism for GTs and glycosidases trafficking. Arginine and Lysine residues are commonly found in most GTs and glycosidases and can directly interact with cargo receptors and COP coatomers. The positive charge and branched structure of the arginine and lysine residues are critical in the protein–protein interactions with the cargo receptors and COP coatomers. Some motifs are the ER-retrieval signal and Golgi-retrieval signal, but the mechanism of recognizing and distinguishing these two signaling sequences remains unclear. One possible mechanism might depend on the different positions of these motifs in the structure of GTs and glycosidases, determining the specific type of the retrieval signal. Different isoforms of COP coatomers and cargo receptors recognize these motifs at various positions. For the GTs that lack the motifs and specific amino acids recognized by COP coatomers, the protein–protein interaction with other GTs or cargo proteins is critical to their proper localization. These protein complexes work as a unit of cargo in COP-coated cargo carriers. In addition, the localization and transportation of GTs are regulated by various inhibitors and environmental conditions in the ER/Golgi. The altered distribution of GTs influences the outcome of glycosylation in the ER and Golgi, and ultimately, signal diverse pathways, affecting cell development and growth. In different types of cells, the mechanism for trafficking the identical or homologous GTs can be numerous, affecting the cell differentiation and function differently. As a whole, the proper localization and effective trafficking of GTs and glycosidases are the prerequisites of their proper and efficient functioning. They require broader and intensive investigation to advance our knowledge in this significant field of research.

## Figures and Tables

**Table 1:** The cargo sorting signals of GTs and glycosidases in their trafficking via COP-dependent transportation.

COPI-Dependent Transportation					
Motif	Species	Interaction with	Position	GTs	Ref.
(F/L)-(L/I/V)-X-X-(R/K)	Yeast	Vps74p	Cytosolic tail	Kre2, Mnn5, Mnn9, Mnn2, Ktr6	[96]
RRXXXXR	Mouse	β-and/or δ-COP (data not shown)	Cytosolic tail	M1-SAT-I	[100]
Di-arginine motifs/lumen domain	*A.thaliana*	Receptor (putative)	Cytosolic tail/ lumen domain	AtGCSI	[98]
ϕ- (K/R)-X-L-X-(K/R)	Human	β -, ζ-COP and MHD domain of δ-COP	Cytosolic tail	Ptase, C2GNT1, GALNT3, GALNT8	[103]
ϕ- (K/R)-X-L-X-(K/R)	Human	β -COP and MHD domain of δ-COP	Cytosolic tail	GALNT6	[103]
WTW	Human	β -COP and MHD domain of δ-COP	Cytosolic tail	GALNT4	[103]
Q residue	*N. benthamiana/ A.thaliana*		TMD	GnTI	[105,106]
	Human	GOLPH3		SiaTI, C2GnT	[110]
**COPII-Dependent Transportation**					
Motif	Species	Interaction with	Position	GTs	Ref.
RPWS/ first two TMDs	Human	Sar1 (putative)	Cytosolic tail/ TMDs	UBIAD1	[104]
[RK](X)[RK]	Mouse/ Human	Sar1 Sec23p	Cytosolic tail	GalT2, GalNAcT	[102]
RLR	Rat		Cytosolic tail	β1,4GT	[102]
RR, RTR	Chicken		Cytosolic tail	Sial-T2	[102]
R and K residues	*N. tabacum/A.thaliana*		Cytosolic tail	GnTI, XylT, GMII	[101]
Di-leucine motif /[RK]X[RK] motifs	Human		*N*-terminus/C-terminus	Precursor protein of α/β-subunit of PT complex	[108]
**Others**					
Motif	Species	Interaction with	Position	GTs	Ref.
LPYS	*A.thaliana*		Cytosolic tail	MNS3	[97]
Cytosolic tail or luminal stem and TMDs	Human			GalNAc-T7	[107]
Luminal stem and TMDs	Human			GalNAc-T10	[107]
Cytosolic tail and TMDs	Human			GalNAc-T1, GalNAc-T2	[107]

## Data Availability

Not applicable.
